# Effects of Drying Process on the Volatile and Non-Volatile Flavor Compounds of *Lentinula edodes*

**DOI:** 10.3390/foods10112836

**Published:** 2021-11-17

**Authors:** Lijia Zhang, Xiaobo Dong, Xi Feng, Salam A. Ibrahim, Wen Huang, Ying Liu

**Affiliations:** 1 College of Food Science and Technology, Huazhong Agricultural University, Wuhan 430070, China; 13297021290@163.com (L.Z.); xbdong@nwafu.edu.cn (X.D.); huangwen@mail.hzau.edu.cn (W.H.); 2 College of Food Science and Engineering, Northwest A&F University, Xianyang 712100, China; 3 Department of Nutrition, Food Science and Packaging, San Jose State University, San Jose, CA 95192, USA; xi.feng@sjsu.edu; 4 Department of Family and Consumer Sciences, North Carolina A&T State University, 171 Carver Hall, Greensboro, NC 27411, USA; ibrah001@ncat.edu

**Keywords:** *Lentinula edodes*, drying methods, volatile compounds, non-volatile compounds, sulfur compounds, free amino acids, 5′-nucleotide

## Abstract

In this study, fresh *Lentinula edodes* was dehydrated using freeze-drying (FD), hot-air drying (HAD), and natural drying (ND), and the volatile and non-volatile flavor compounds were analyzed. The drying process changed the contents of eight-carbon compounds and resulted in a weaker “mushroom flavor” for dried *L. edodes*. HAD mushrooms had higher levels of cyclic sulfur compounds (56.55 μg/g) and showed a stronger typical shiitake mushroom aroma than those of fresh (7.24 μg/g), ND (0.04 μg/g), and FD mushrooms (3.90 μg/g). The levels of 5′-nucleotide increased, whereas the levels of organic acids and free amino acids decreased after the drying process. The dried *L. edodes* treated with FD had the lowest levels of total free amino acids (29.13 mg/g). However, it had the highest levels of umami taste amino acids (3.97 mg/g), bitter taste amino acids (6.28 mg/g) and equivalent umami concentration (EUC) value (29.88 g monosodium glutamate (MSG) per 100 g). The results indicated that FD was an effective drying method to produce umami flavor in dried mushrooms. Meanwhile, HAD can be used to produce a typical shiitake mushroom aroma. Our results provide a theoretical basis to manufacture *L. edodes* products with a desirable flavor for daily cuisine or in a processed form.

## 1. Introduction

*Lentinula edodes* (Berk.) Sing or Shiitake mushroom, accounting for about 17% of global mushroom production, is the second largest cultivated edible mushroom in the world [1,2]. The global production of cultivated mushrooms has increased more than 73% in the last 10 years (FAOSTAT, 2019). *L. edodes* is one of the most popular edible and medicinal mushrooms in East Asia for its rich nutrients, favorable medicinal properties as well as desirable flavor [3,4]. Shiitake mushroom has been widely used as a flavor enhancer in meat and fermented products due to its unique flavor [5,6].

The desirable flavor of shiitake mushrooms is composed of volatile and non-volatile components [3]. It has been identified with various volatile compounds from *L. edodes*. The major volatile components are eight-carbon (C8) compounds and sulfur compounds [1]. Both C8 and sulfur compounds are produced by enzymatic reactions, in which lipoxygenase and hydroperoxide lyase catalyze linoleic acid to produce C8 compounds, such as 1-octen-3-one and 1-octen-3-ol, whereas γ-glutamyl transpeptidase and cysteine sulfoxide lyase catalyze lenthinic acid to produce sulfur compounds, including 1,2,4-trithiolane and lenthionine [7,8]. Among these volatile compounds, 1-octen-3-ol, widely present in mushrooms, is responsible for a typical mushroom-like odor, whereas lenthionine, a cyclic 5-sulfur compound, also contributes to the aroma of *L. edodes* [9,10]. Fresh shiitake mushroom gives off a slight odor by the abundance of 1-octen-3-ol. However, the unique aroma of shiitake mushroom develops due to the increase of sulfur compounds after drying [1]. The taste of mushrooms is mainly triggered by several small water-soluble substances, including organic acids, 5′-nucleotides and free amino acids [11]. Thus, it is vital to investigate the effects of processing methods on volatile and non-volatile compounds of shiitake mushrooms.

Drying is a convenient and effective technique to prolong the shelf life of fresh shiitake mushrooms by decreasing the moisture content [12,13]. However, the quality of mushrooms (flavor, nutrients, texture and color) is affected by the drying method [12]. Hot-air drying (HAD), freeze-drying (FD) and natural drying (ND) are the three most typical drying methods for shiitake mushrooms. Lu et al. [14] studied the changes of volatile components in *L. edodes* during the vacuum freeze-drying process. Qin et al. [15] evaluated the changes in aroma profile of *L. edodes* during different stages of HAD. Politowicz et al. [12] investigated the effects of different drying methods (CD (convective drying), FD (freeze-drying), VMD (vacuum-microwave drying) and CPD-VMFD (vacuum–microwave finish-drying)) on volatile composition and sensory profile (inner color and sponginess) of *L. edodes*. However, the influence of the drying methods of hot-air drying (HAD), freeze drying (FD) and natural drying (ND) on both the volatile and non-volatile flavor compounds in *L. edodes* has never been studied. 

The objectives of this work were to analyze the volatile and non-volatile flavor components’ profile of *Lentinula edodes* by three drying methods (HAD, FD and ND). The results could provide theoretical evidence for new product developments with *Lentinula edodes*.

## 2. Materials and Methods

### 2.1. Material

Fresh shiitake mushrooms were obtained from a local market (Wuhan, China). The samples with uniform size (including the cap and stipe) and maturity were selected. The moisture contents of shiitake mushrooms were 87.50 ± 1.30% (g/g, w.b.). Internal standard solution (cyclohexanone (99.50% purity)) was purchased from FLUKA (Seelze, Germany). The n-alkane standards (C_7_–C_30_) were purchased from Sigma Chemical Company (St. Louis, MO, USA).

### 2.2. Drying Methods

#### 2.2.1. Freeze-Drying (FD)

Fresh shiitake mushrooms (1000 g) were dried by a freeze drier (Betr 2–8 LD plus, Christ, Germany). The vacuum degree was 20–40 Pa. Cold trap temperature was −50 °C. The drying chamber was −35 °C. Drying was carried out for 72 h until the moisture content was less than 10% (g/g, w.b.) [11].

#### 2.2.2. Hot Air Drying (HAD)

Fresh shiitake mushrooms (1000 g) were directly dried in a forced air circulation oven (GZX-9240MBE, Yuhua Equipment Ltd., Gongyi, China). The temperature was set at 50 °C and the air velocity was at 0.45 m/s. Samples were dried until the final moisture content was below 10% (g/g, w.b.). 

#### 2.2.3. Natural Drying (ND)

Fresh shiitake mushrooms (1000 g) were placed on trays in a single layer. Samples were then placed outdoors in a well-ventilated location with abundant sunlight. The average temperature was 25 ± 5 °C and the relative humidity was 70 ± 10%. The drying lasted for about 3 days until the final moisture content was below 10% (g/g, w.b.).

### 2.3. Electronic Nose Analysis

An FOX 4000 electronic nose system from Alpha M.O.S. (Toulouse, France) was used for electronic nose analysis [16]. The instrument comprised of 18 metal oxide sensors and combined with a headspace auto-sampler HS100. Dried shiitake mushroom samples were ground to a fine powder and screened by a 60-meshes sifter. Then, fresh mushroom and different dried powders were homogenized in saturated sodium chloride solution (1:20), respectively. Sample of 2 mL were added into a 10 mL vial and capped with a Teflon rubber cap, equilibrated at 40 °C for 120 s under agitation (500 rpm). Dry air was used at a flow of 150 mL/min as carrier gas. Equilibrated headspace (2500 µL) was injected into the electronic nose at the rate of 2500 µL/s by a 2500 µL gas-tight syringe (50 °C). The acquisition time and delay time between consecutive injections were set as 120 s and 300 s, respectively.

### 2.4. HS-SPME-GC-MS Analysis

The volatile compounds were analyzed following Jing-Nan et al.’s methods with a minor modification [17]. An SPME (solid-phase micro-extraction) manual device equipped with a 50 µm/30 µm divinylbenzene/carboxen/polydimethylsiloxane (DVB/CAR/PDMS) fiber (Supelco, Bellfonte, PA, USA) was used to extract volatile compounds from samples. Mushroom homogenate (1.8 g mushroom powder in 20 mL NaCl saturated solution) was added into 40 mL-vial containing a magnetic stirring bar. After being spiked with 25 µL of cyclohexanone (0.95 mg/mL of ethyl alcohol) as internal standard, the vials were immediately seal with a PTFE septa (Supelco, Bellfonte, PA, USA). Samples were balanced at 50 °C for 15 min, then the fiber was inserted into the vial to extract volatile compounds for 1 h. Finally, the fiber was inserted into the injection port of GC and desorbed for 5 min under the splitless mode.

An Agilent 7890A GC coupled with an Agilent 5975 MS was used to analyze volatile compounds. A HP-5MS fused silica capillary column (30 m × 0.25 mm I.D., 0.25 mm film thickness, Agilent technologies, Santa Clara, CA, USA) was installed in GC. GC conditions were set as follows: the carrier gas of helium with a flow rate of 1 mL/min; the injector temperature was 250 °C; the oven temperature programming was set at 40 °C for 3 min initially, then 3 °C/min to 150 °C, held for 1 min, finally 5 °C/min to 220 °C, maintained for 2 min. The ionization source temperature was set at 230 °C. The MS was obtained by electron impact mode at 70 eV in a range from 30 amu to 395 amu.

Volatile compounds were identified by Kovats retention index (RI) and the database (Wiley7.0 and NIST05) [15]. The Kovats retention index (RI) of unknown compounds was calculated using n-alkanes (C7-C30) injected under the same conditions. The quantity of volatiles was calculated with the internal standard (cyclohexanone) [18].

### 2.5. Assay of Organic Acids

Organic acids were extracted and analyzed as the cited method [19]. A sample (500 mg) was used to extract organic acids with 25 mL of KH_2_PO_4_ (pH = 2.65) in 75 °C water bath for 25 min. The solution was centrifuged 20 min at 10,000 rpm, then filtered and analyzed by HPLC system. The HPLC system is equipped with an InertSustain AQ-C18 column (4.6 × 250 mm, 5 μm) (Shimadzu, Shanghai, China). Dipotassium phosphate (0.01 mol/L, pH = 2.65) was used as mobile phase with a flow rate at 0.4 mL/min. The injection volume was 20 µL and wavelength of the UV detector was 214 nm. Organic acids in the samples were identified by the retention time of the standard organic acids (Sinopharm Chemical Reagent Co. Ltd., Shanghai, China) and quantified with their standard curves.

### 2.6. Assay of Free Amino Acids

According to the report [11], 1 g of mushroom powder and extract was obtained with 50 mL of hydrochloric acid (0.10 mol/L) at 25 °C for 45 min. Then, centrifuged for 30 min at 12,000 rpm. The free amino acids were analyzed by an L-8900 high-speed amino acid analyzer (Hitachi High-Tech. Corp., Tokio, Japan).

### 2.7. Assay of 5′-Nucleotides

5′-nucleotides was extracted and analyzed following the cited method [11]. Sample power (500 mg) was used to extract 5′-nucleotides with distilled water (25 mL) at 100 ± 5 °C for 1 min. After centrifugation (10,000 rpm, 15 min), the supernatant was evaporated. Then, the residue was re-dissolved in deionized water (10 mL). Sample of 20 µL was injected to the same HPLC system as described in Section 2.5. 5′-nucleotide in the samples were identified by the retention time of the standard 5′-nucleotide (Sigma, USA) and quantified with their standard curves.

### 2.8. Statistical Analysis

Principal component analysis (PCA) was performed by XLSTAT 2010 (Microsoft Corporation, Washington, DC, USA). One way analysis of variance (ANOVA) was used to analyze the differences among samples at a significant level of 0.05 by SPSS 25 (IBM, Armonk, NY, USA).

## 3. Results and Discussion

### 3.1. Electronic Nose Analysis of L. edodes Samples

**Figure 1:**
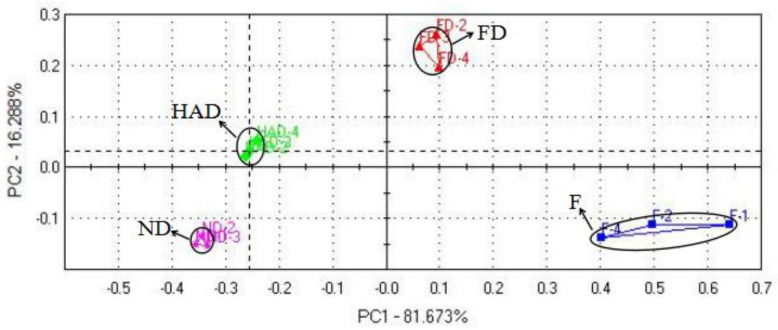
PCA of E-nose data for fresh and dried *L. edodes*; F: fresh sample; FD: freeze drying sample; HAD: hot-air drying sample; ND: natural drying sample.

The electronic nose obtains comprehensive flavor information within a short time by mimicking the human olfactory system [20]. It was used for classifying and monitoring the drying process of *L. edodes* [14,21]. Principal component analysis (PCA) is a multivariate chemometric method that can be used to identify the correlation patterns of constituent variables involved in the distinction between samples [16]. PCA also can provide a better visualization and highlight the differences in volatile profiles. As shown in Figure 1, the accumulative variance contribution rate of the first two PCs was 93% (PC1 accounted for 81.67% and PC2 accounted for 16.29%), which indicated the feasibility of PCA and the two main components contained most of the information about volatile compounds [22]. Despite sensor instability among replications, volatile profiles from different samples were located into four separated areas. FD samples located in the positive axis of PC1, while both ND and HAD samples were in the negative axis of PC1, which indicated that the volatile compounds of ND and HAD samples were significantly different from those of FD samples. These results also showed that the drying processes altered shiitake mushroom aroma profiles.

### 3.2. HS-SPME-GC-MS Analysis of Volatile Compounds of L. edodes

In order to further investigate the effects of different drying methods on the flavor of *L. edodes*, the specific volatile components of different mushroom samples were measured by HS-SPME-GC-MS. Semi-quantitative analysis with an internal standard was used to compare differences among samples. The volatile compounds of fresh and dried mushroom samples were shown in Table 1. A total of 55 volatile compounds from *L. edodes* were tentatively identified and quantified, including 11 alcohols, 9 aldehydes, 6 ketones, 11 sulfur compounds and 17 hydrocarbons. A total of 41, 36 and 24 compounds were detected in FD, HAD and ND samples, with contents of 171.29 µg/g, 206.88 µg/g and 256.67 µg/g, respectively. It is notable that only 22 compounds were detected in fresh mushroom (1451 µg/g). It was consistent with the reports that more volatile compounds were formed in dried *L. edodes* [1]. After drying treatment, the total volatile components increased, but the total volatile compounds content decreased.

**Figure 2:**
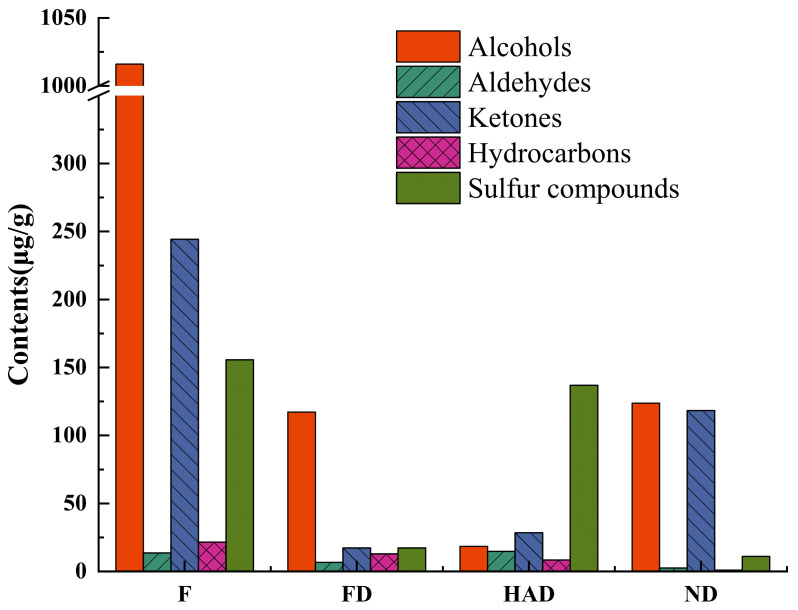
Contents of different types of volatile compounds in fresh and different dried *L. edodes*; F: fresh sample; FD: freeze drying sample; HAD: hot-air drying sample; ND: natural drying sample.

It has been reported that alcohols, aldehydes and ketones played important roles in flavor profiles of foods [9,10,23,24]. As shown in Table 1, the contents of alcohols, aldehydes and ketones in fresh, FD, HAD and ND were 1273.89 µg/g, 141.14 µg/g, 61.58 µg/g, and 244.74 µg/g, respectively. However, the contents of alcohols and ketones significantly decreased after the drying process. Similar results were reported in other mushrooms [9,10]. As shown in Figure 2, alcohols were the highest in fresh samples, but decreases were shown, especially in HAD mushroom samples. The content of aldehydes in HAD mushroom samples increased slightly in comparison with fresh samples. This may be due to aldehydes mainly derived from the oxidation and degradation of unsaturated fatty acids [25]. In addition, increasing the temperature of *L. edodes* samples may also promote Maillard reaction, including Strecker degradation, which increased aldehyde content [26]. The content of ketones in FD samples was the lowest. It was reported that ketones are mainly obtained from the Maillard reaction and the oxidation and degradation of unsaturated fatty acids [27], and ketones can also be produced by degradation of esters [28]. However, in this study, the most probable route may be through enzymatic reactions. The preservation or formation of ketones in *L. edodes* was not conducive when the FD temperature was not appropriate [14].

The flavor differences between fresh and dried *L. edodes* were obvious. The “mushroom flavor” of fresh *L. edodes* is described as a sweet, earthy and cheesy aroma, while the shiitake flavor of dried *L. edodes* is similar to that of onion and garlic [29]. Eight-carbon compounds are ubiquitous among mushrooms, which are a key contributor to mushroom flavor [9,30]. As shown in Table 1, the contents of eight-carbon compounds in fresh, FD, HAD, and ND mushrooms were 1271.85 µg/g, 131.18 µg/g, 38.89 µg/g and 242.69 µg/g, respectively. The contents of 1-octen-3-ol in in fresh, FD, HAD, and ND mushrooms were 857.02 µg/g, 103.78 µg/g, 5.72 µg/g and 105.30 µg/g, respectively. The contents of 1-octen-3-one and 3-octanone in in fresh, FD, HAD, and ND mushrooms were 244.08 µg/g, 17.13 µg/g, 27.96 µg/g and 118.24 µg/g, respectively. Generally, 1-octen-3-ol, 1-octen-3-one and 3-octanone were the major C8 compounds in mushrooms. 1-octen-3-ol, also called mushroom alcohol, feature with a typical odor of mushrooms [29]. 1-octen-3-one and 3-octanone could produce a similar odor like 1-octen-3-ol [31]. As shown in Table 1, 1-octen-3-ol and 1-octen-3-one were detected in all of the four mushrooms, and fresh mushrooms had the highest level of 1-octen-3-ol and 1-octen-3-one. 3-octanone was detected in fresh, FD, and HAD mushrooms, and FD mushrooms had the highest amount. However, other research reported that 1-octen-3-ol and 3-octanone were the dominant compounds in the dried *L. edodes* (vacuum, microwave, microwave vacuum, convective drying, vacuum-microwave drying and freeze-drying) [1,12]. The differences were possibly due to the different volatile compound extraction methods. In sum, drying processes changed the contents of C8 compounds, which resulted in weaker “mushroom flavor” for dried *L. edodes* compared with fresh *L. edodes*. This interesting finding could be helpful to further understanded the biosynthesis of C8 compounds in mushrooms.

Volatile sulfur compounds are important aroma-active compounds in vegetables and processed meats, and they can offer unique aroma in *L. edodes* differing from many other commonly consumed mushrooms [22,32]. As shown in Table 1, the contents of sulfur compounds in mushrooms decreased after the drying process. However, more types of sulfur compounds were detected from dried mushrooms compared with fresh mushrooms. which was similar to that reported by Lu et al. [14]. Carbon disulfide from mushrooms had little influence in relation to the overall aroma [33]. The straight chain sulfur compounds and cyclic sulfur compounds were reported to greatly contribute to the unique aroma of *L. edodes* [34]. As shown in Table 1, the straight chain sulfur compounds, dimethyl disulfide and dimethyl trisulfide, were not detected in fresh and ND mushrooms, while dimethyl disulfide was detected in HAD mushrooms, and dimethyl trisulfide was detected in both FD and HAD mushrooms. The results showed that 1,2,4-trithiolane, 1,2,4,5-tetrathiane and lenthionine were the main cyclic sulfur compounds detected in the four kinds of mushroom samples. HAD mushrooms had the highest level of cyclic sulfur compounds (56.55 μg/g) among the four mushroom samples, and the content of cyclic sulfur compounds in fresh shiitake mushroom, FD and ND were 7.24 μg/g, 3.90 μg/g and 0.04 μg/g, respectively. Tian et al. [1] reported that HAD increased the content of cyclic sulfur compounds compared with fresh shiitake mushrooms. The formation of these cyclic sulfur compounds included two steps: the enzymic reactions of lenthinic acid catalyzed by cysteine sulfoxide lyase and γ-glutamyl transpeptidase; and the nonenzymatic polymerization of methylene disulfide [8]. In addition, lenthionine can decompose to dimethyl disulfide and dimethyl trisulfide [35]. Therefore, the differences of sulfur compounds in FD, HAD, and ND mushrooms might be attributed to enzyme activities and drying temperature during drying processes.

In sum, different drying processes significantly affected the volatile profiles of *L. edodes*, especially the C8 and sulfur compounds. HAD mushrooms had weaker “mushroom flavor” and strong shiitake flavor. In fact, the volatile content of *L. edodes* was different in each stage (fresh, early, middle and late stage) of HAD [21]. The above complicated changes of volatile compounds in mushrooms may be attributed to typical drying conditions, such as higher temperature for HAD, long-time exposure in the sunlight for ND and vacuum evaporation for FD, which could affect volatile compounds formation and precursor degradation.

### 3.3. Effects of Drying Methods on Organic Acids of L. edodes

Organic acids (tartaric acid, malic acid, ascorbic acid, citric acid, fumaric acid and succinic acid) were detected (Table 2). As shown in Table 2, succinic acid (472.61–645.25 mg/g) was the most abundant organic acid in the samples, accounting for more than 70% of the organic acid content, followed by citric acid (107.65–142.31 mg/g) and malic acid (21.00–36.22 mg/g). Chen et al. [36] reported that the content of main organic acids was highest, in descending order, with succinic acid, citric acid and malic acid in *L. edodes*. These results were consistent with our results. The total content of organic acids in fresh and different dried *L. edodes* ranged from 621.32 mg/g to 875.82 mg/g and were in the descending order of fresh (875.82 mg/g), ND (695.30 mg/g), HAD (667.82 mg/g), FD (621.32 mg/g). The result was higher than that of the organic acids in other species (*Agrocybe cylindracea*, *Pleurotus cystidiosus*, *Agaricus blazei*, *Pleurotus eryngii*, *Coprinus comatus*), which ranged from 59.42 mg/g in to 237.81 mg/g [19]. However, compared with total organic acids of fresh *L. edodes*, the drying process decreased the relative contents, and FD samples were the lowest.

### 3.4. Effects of Drying Methods on Free amino Acids of L. edodes

*Lentinus edodes* contains a variety of amino acids. They can provide strong umami and pleasant sweet flavors [37]. As shown in Table 3, the contents of total free amino acids in fresh and dried *L. edodes* ranged from 29.13 to 32.82 mg/g dry weight. The total free amino acids content of the fresh samples was 32.817 mg/g dry weight, which was consistent with the reported value (31.70 mg/g) [38]. The content of total free amino acids in FD samples was the lowest. This may be due to the high temperature promoting protein hydrolysis.

**Figure 3:**
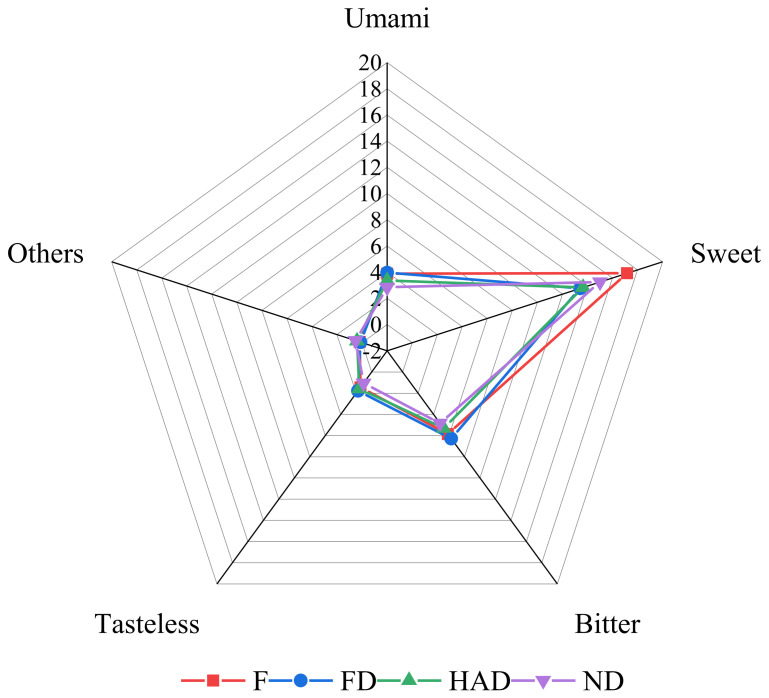
Radar graph of the sensory results of fresh and different dried *L. edodes*; F: fresh sample; FD: freeze drying sample; HAD: hot-air drying sample; ND: natural drying sample; Uma-mi = ASP + GLU; Sweet = Ala + Gly + Ser + Thr; Bitter = Arg + His + Ile + Leu + Met + Phe + Val; Tasteless = Lys + T-yr; Other = GABA + Orn.

Free amino acids were classified into umami, sweet, bitter and tasteless based on their taste properties [39]. As shown in Table 3, threonine was the highest sweet amino acid, accounting for 77% to 87%, while glutamic acid was the highest umami taste amino acid. The results are similar to others [36]. The contents of aspartic acid were significantly increased in FD samples compared with fresh, HAD and ND. As revealed in Figure 3, fresh *Lentinus edodes* contained the sweetest amino acid taste, while the FD sample was the most umami-like and bitter tasting amino acid.

### 3.5. Effects of Drying Methods on 5′-Nucleotides of L. edodes

5′-nucleotides in mushrooms contribute to umami taste [1]. 5′-nucleotides (5′-CMP, 5′-UMP, 5′-GMP, 5′-AMP) were detected in this study (Table 4). As shown in Table 4, the total content of 5′-nucleotides in fresh and different dried *L. edodes* ranged from 7.94 to 14.41 mg/g, which indicated the 5′-nucleotide increased after drying. In addition, 5′-CMP (3.14–8.36 mg/g) and 5′-AMP (2.60–4.92 mg/g) were found as the main 5′-nucleotide in *L. edodes* samples.

### 3.6. Equivalent Umami Analysis

**Figure 4:**
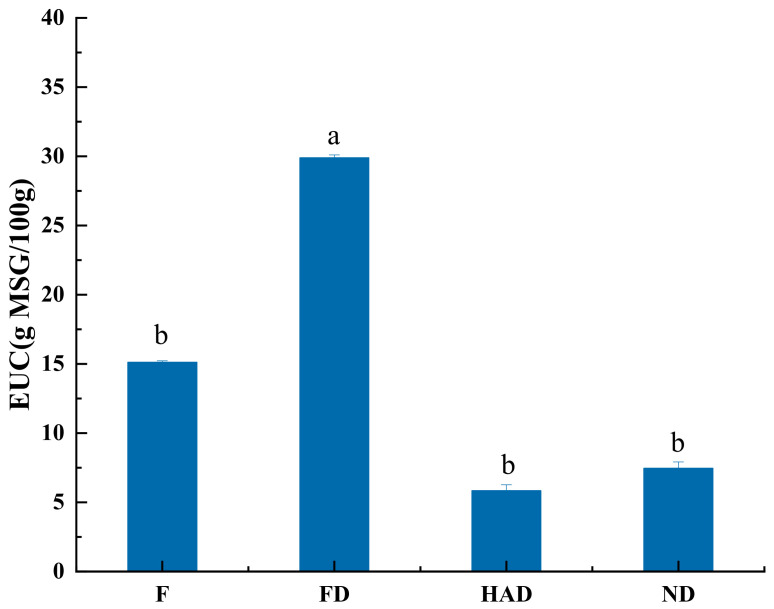
Equivalent umami concentration (EUC) values of fresh and different dried *L. edodes*; F: fresh sample; FD: freeze drying sample; HAD: hot-air drying sample; ND: natural drying sample. Means with different letters (a, b) are significantly different (*p* < 0.05).

The equivalent umami concentration (EUC) represents the MSG concentration, based on the synergistic effect of MSG-like components (Asp and Glu) and 5′-nucleotide (5′-AMP, 5′-GMP and 5′-IMP), which may enhance the umami taste of mushrooms [3]. As shown in Figure 4, the EUC value of fresh and different dried *L. edodes* ranged from 5.84 to 29.88 g MSG/100 g, which were similar to the reported results [19]. It is reported that the EUC values defined at four levels: <10% (<10 g MSG/100 g dry matter), 10–100% (10–100 g MSG/100 g), 100–1000% (100–1000 g MSG/100 g) and >1000% (>1000 g MSG/100 g) [40]. The EUC values of the HAD and ND samples at the first level (<10 g MSG/100 g), fresh and FD samples at the second level (10–100 g MSG/100 g) (Figure 4). Among the four samples, the FD dried *L. edodes* had the highest EUC value (29.88 g MSG/100 g) and were in the descending order of FD > fresh (15.12 g MSG/100 g) > ND (7.46 g MSG/100 g) > HAD (5.84 g MSG/100 g), which proved FD as an effective drying method to produce umami tasting dried *L. edodes*.

## 4. Conclusions

In the present study, volatile and non-volatile compound profiles of *L*. *edodes* were modified by three typical drying methods (FD, HAD and ND). After drying, the content of C8 volatile compounds decreased, but sulfur compounds (straight chain and cyclic sulfurs) were increased, especially in the HAD mushrooms, which resulted in strong shiitake flavor. The drying process increased the relative contents of total 5′-nucleotide of *L. edodes* as well as the freeze-drying *L. edodes* had the highest levels of umami taste amino acids, bitter taste amino acids and EUC value. Hot-air drying would be a better method to produce typical shiitake mushroom aroma, while FD was an effective drying method to produce umami tasting dried *L. edodes*.

The growth of the global dried mushroom market is driven by the demands of organic and healthy food with a clean label. In order to provide nutrient and high-quality final products to consumers, fundamental research should be conducted to better understand the mechanisms of macro and micro-nutrient changes and flavor formation during processing. This research provided up-to-date information about the formation of volatile and non-volatile compounds during different drying processing, which could provide useful information to the mushroom industry to select the optimum method to process dried mushrooms without any preservatives and additives. Future research will focus on understanding the contribution of volatile and non-volatile compounds to sensory perception.

## Figures and Tables

**Table 1:** Contents of volatile compounds in fresh and different dried *L. edodes*.

Compounds		RI ^a^	Contents (μg/g)				ID ^e^
			F ^b^	FD	HAD	ND	
**Alcohols (11)**	1-Hexanol	871	nd ^d^	0.40	0.56	1.17	A
	1-Octen-3-ol ^c^	983	857.02	103.78	5.72	105.30	B
	2-Cyclohexen-1-ol	985	nd	nd	7.22	nd	A
	3-Octanol ^c^	998	6.09	1.93	nd	1.40	B
	2-Octen-1-ol ^c^	1071	132.99	6.35	2.59	13.86	B
	1-Octanol ^c^	1077	19.89	1.99	0.68	2.06	B
	Linalool	1100	nd	2.13	nd	nd	A
	Phenylethyl Alcohol	1117	nd	0.08	0.95	nd	A
	4-Methyl-benzeneethanol	1173	nd	nd	0.66	nd	A
	α-Terpineol	1188	nd	0.43	nd	nd	B
	Cedrol	1596	nd	0.11	nd	nd	B
	***Total alcohols***		1015.99	117.20	18.39	123.79	
**Aldehydes (9)**	Hexanal	803	nd	0.34	4.62	0.02	A
	(E)-Hept-2-enal	958	nd	nd	nd	0.18	A
	Benzaldehyde	965	0.69	0.62	1.30	0.15	A
	Octanal ^c^	1004	nd	nd	0.18	nd	A
	Benzeneacetaldehyde	1046	0.48	3.44	0.93	0.32	A
	(E)-2-Octenal ^c^	1058	11.78	nd	1.76	1.72	A
	2-Phenylpropenal	1157	nd	0.67	5.64	0.07	A
	Decanal	1206	0.69	0.42	0.27	0.09	A
	(z)-3,7-Dimethylocta-2,6-dienal	1241	nd	1.18	nd	nd	A
	***Total aldehydes***		13.64	6.67	14.70	2.55	
**Ketones (6)**	1-Octen-3-one ^c^	981	237.99	5.52	21.17	118.24	B
	3-Octanone ^c^	991	6.09	11.61	6.79	nd	B
	3-Octen-2-one ^c^	1042	nd	nd	nd	0.11	A
	Acetophenone	1066	nd	nd	0.14	0.02	A
	(+)-Camphor	1139	0.18	0.08	0.05	0.02	B
	2-Undecanone	1297	nd	0.06	0.34	0.01	A
	***Total ketones***		244.26	17.27	28.49	118.40	
**Sulfur compounds (11)**	Carbon disulfide	<700	148.36	12.51	66.31	11.01	A
	Dimethyl disulfide	700	nd	nd	2.58	nd	B
	Dimethyl trisulfide	973	nd	0.80	0.26	nd	B
	1,2,4-Trithiolane	1083	7.00	2.79	32.21	nd	B
	2,4,5-Trithiahexane	1121	nd	nd	4.87	nd	A
	Dimethyl tetrasulfide	1209	nd	nd	2.27	nd	A
	1,2,4,5-Tetrathiane	1362	nd	0.11	7.23	nd	B
	1,2,4,6-Tetrathiepane	1504	nd	0.01	3.22	nd	B
	Lenthionine	1615	0.24	1.00	17.11	0.04	B
	Hexathiepane	1615	nd	nd	0.25	nd	B
	Cyclic octaatomic sulfur	2000	nd	nd	0.29	nd	A
	***Total sulfur compounds***		155.60	17.22	136.60	11.05	
**Hydrocarbons (17)**	1,3-Xylene	865	nd	0.05	0.04	0.03	A
	P-Isopropyltoluene	1021	1.06	0.32	nd	0.03	A
	D-Limonene	1025	17.43	10.54	7.74	0.78	A
	Naphthalene	1176	0.30	0.33	0.54	0.02	A
	Dodecane	1199	0.16	0.38	nd	nd	A
	5-Ethyl-2-methyl-octane	1280	0.95	nd	nd	nd	A
	3-Carene	1283	nd	0.07	nd	nd	B
	Decane	1288	nd	0.13	nd	nd	A
	b-Elemen	1390	nd	0.08	nd	nd	A
	Tetradecane	1400	0.56	0.14	0.09	0.00	A
	Germacrene D	1477	nd	0.08	nd	nd	A
	(-)-a-Selinenea-	1491	nd	0.20	nd	nd	A
	Pentadecane	1501	0.91	0.39	nd	nd	A
	Hexadecane	1600	nd	0.11	0.05	0.02	A
	Heptadecane	1697	nd	0.05	nd	nd	A
	Eicosane	2000	0.14	0.06	nd	nd	A
	***Total hydrocarbons***		21.51	12.93	8.46	0.88	

RI ^a^, retention index. F ^b^, fresh shiitake mushroom; FD, freeze drying sample; HAD, hot-air drying sample; ND, natural drying sample. eight-carbon ^c^ compounds. nd ^d^, not detected. Identification ^e^: A, a comparison of mass spectrum and RI with authentic standards; B, comparison of mass spectrum and RI with published data and Wiley 7.0 and NIST05 MS library.

**Table 2:** Organic acid contents of fresh and different dried *L. edodes*.

Organic Acids (mg/g)	F ^a^	FD	HAD	ND
Tartaric acid	41.91 ± 1.97 ^A^	Nd ^b^	12.18 ± 0.19 ^B^	Nd ^b^
Malic acid	36.22 ± 0.70 ^A^	27.82 ± 0.77 ^B^	23.57 ± 2.35 ^BC^	21.00 ± 0.38 ^C^
Ascorbic acid	6.27 ± 0.28 ^A^	5.00 ± 0.27 ^C^	5.66 ± 0.11 ^B^	4.88 ± 0.18 ^C^
Citric acid	134.49 ± 0.45 ^C^	107.65 ± 2.17 ^D^	138.13 ± 1.90 ^B^	142.31 ± 1.09 ^A^
Fumaric acid	11.68 ± 0.03 ^A^	8.23 ± 0.03 ^B^	6.57 ± 0.02 ^D^	7.61 ± 0.02 ^C^
Succinic acid	645.25 ± 3.03 ^A^	472.61 ± 3.34 ^D^	481.71 ± 1.72 ^C^	519.51 ± 2.11 ^B^
Total	875.82 ± 2.04 ^A^	621.32 ± 3.42 ^D^	667.82 ± 0.48 ^C^	695.30 ± 0.96 ^B^

Mean ± SD (*n* = 3). Means with different superscript (A, B, C, D) in the same row are significantly different (*p* < 0.05). F ^a^, fresh shiitake mushroom; FD, freeze drying sample; HAD, hot-air drying sample; ND, natural drying sample. nd ^b^, not detected.

**Table 3:** The content of free amino acids in fresh and different dried *L. edodes*.

Free Amino Acids (mg/g)		F ^a^	FD	HAD	ND
Umami Taste Amino Acids	Asp ^b^	0.56 ± 0.01 ^B^	1.57 ± 0.001 ^A^	0.10 ± 0.01 ^C^	0.10 ± 0.001 ^C^
	Glu	3.33 ± 0.02 ^A^	2.40 ± 0.001 ^C^	3.26 ± 0.01 ^A^	2.76 ± 0.003 ^B^
	total	3.89 ± 0.02 ^AB^	3.97 ± 0.001 ^A^	3.36 ± 0.02 ^AB^	2.86 ± 0.003 ^B^
	Ala	0.74 ± 0.003 ^D^	1.27 ± 0.003 ^B^	1.80 ± 0.01 ^A^	0.95 ± 0.003 ^C^
Sweet Taste Amino Acids	Gly	0.77 ± 0.003 ^A^	0.85 ± 0.001 ^A^	0.80 ± 0.01 ^A^	0.57 ± 0.003 ^B^
	Ser	0.76 ± 0.01 ^A^	0.72 ± 0.003 ^A^	0.46 ± 0.003 ^B^	0.39 ± 0.003 ^C^
	Thr	14.89 ± 0.01 ^A^	10.57 ± 0.05 ^B^	10.59 ± 0.07 ^AB^	13.10 ± 0.08 ^A^
	total	17.16 ± 0.02 ^A^	13.41 ± 0.06 ^B^	13.65 ± 0.08 ^AB^	15.00 ± 0.07 ^A^
	Arg	2.77 ± 0.002 ^A^	2.07 ± 0.003 ^C^	1.51 ± 0.01 ^C^	2.45 ± 0.01 ^B^
Bitter Taste Amino Acids	His	0.68 ± 0.001 ^A^	0.55 ± 0.001 ^B^	0.37 ± 0.01 ^C^	0.36 ± 0.001 ^C^
	Ile	0.11 ± 0.001 ^B^	0.43 ± 0.001 ^A^	0.42 ± 0.001 ^A^	0.15 ± 0.001 ^B^
	Leu	0.20 ± 0.001 ^B^	0.71 ± 0.001 ^A^	0.66 ± 0.01 ^AB^	0.14 ± 0.003 ^B^
	Met	0.10 ± 0.001 ^A^	0.08 ± 0.01 ^A^	0.07 ± 0.003 ^A^	0.08 ± 0.01 ^A^
	Phe	0.67 ± 0.09 ^A^	0.79 ± 0.01 ^A^	0.92 ± 0.03 ^A^	0.56 ± 0.01 ^B^
	Val	1.33 ± 0.01 ^B^	1.64 ± 0.003 ^A^	1.36 ± 0.01 ^AB^	1.10 ± 0.003 ^C^
	total	5.85 ± 0.10 ^AB^	6.28 ± 0.02 ^A^	5.30 ± 0.07 ^AB^	4.83 ± 0.02 ^B^
Tasteless Amino Acids	Lys	1.16 ± 0.003 ^B^	1.43 ± 0.003 ^A^	1.17 ± 0.001 ^B^	0.86 ± 0.003 ^C^
	Tyr	0.28 ± 0.01 ^A^	0.34 ± 0.003 ^A^	0.42 ± 0.03 ^A^	0.18 ± 0.02 ^A^
	total	1.44 ± 0.02 ^B^	1.77 ± 0.001 ^A^	1.59 ± 0.03 ^B^	1.04 ± 0.02 ^B^
Others	GABA	0.20 ± 0.001 ^B^	0.13 ± 0.003 ^B^	0.41 ± 0.003 ^A^	0.51 ± 0.001 ^A^
	Orn	4.29 ± 0.01 ^C^	3.57 ± 0.01 ^D^	5.32 ± 0.01 ^B^	6.25 ± 0.01 ^A^
Total Amino Acids		32.81 ± 0.09 ^A^	29.13 ± 0.06 ^C^	29.61 ± 0.08 ^BC^	30.48 ± 0.03 ^B^

Mean ± SD (*n* = 3). Means with different superscript (A, B, C, D) within a row are significantly different (*p* < 0.05). F ^a^, fresh shiitake mushroom; FD, freeze drying sample; HAD, hot-air drying sample; ND, natural drying sample. Asp ^b^, L-Aspartic acid; Glu, L-Glutamic acid; Ala, L-Alanine; Gly, Glycine; Ser, L-Serine; Thr, L-Threonine; Arg, L-Arginine; His, L-Histidine; Ile L-Isoleucine; Leu, L-Leucine; Met, L-Methionine; Phe, L-Phenylalanine; Val, L-Valine; Lys, L-Lysine; Tyr, L-Tyrosine; GABA, γ-Aminobutyric Acid; Orn, L-Ornithine.

**Table 4:** 5′-nucleotides in fresh and different dried *L. edodes*.

5′-Nucleotides ^a^	F ^b^	FD	HAD	ND
5′-CMP	3.14 ± 0.03 ^B^	2.96 ± 0.03 ^B^	7.78 ± 0.03 ^A^	8.38 ± 0.06 ^A^
5′-UMP	0.27 ± 0.02 ^C^	0.39 ± 0.004 ^B^	0.92 ± 0.03 ^B^	1.92 ± 0.09 ^A^
5′-GMP	0.33 ± 0.004 ^A^	0.22 ± 0.003 ^A^	0.39 ± 0.03 ^A^	0.57 ± 0.03 ^A^
5′-AMP	4.25 ± 0.01 ^A^	4.92 ± 0.02 ^A^	2.60 ± 0.01 ^B^	3.69 ± 0.26 ^B^
Flavor 5′-nucleotides ^c^	0.33 ± 0.004 ^A^	0.22 ± 0.003 ^A^	0.39 ± 0.03 ^A^	0.57 ± 0.03 ^A^
MSG-like 5′-nucleotide ^d^	4.58 ± 0.002 ^A^	5.14 ± 0.02 ^A^	2.99 ± 0.02 ^B^	4.26 ± 0.29 ^A^
Total	7.94 ± 0.05 ^B^	8.48 ± 0.00 ^B^	11.61 ± 0.08 ^A^	14.41 ± 0.14 ^A^

Mean ± SD (*n* = 3). Means with different superscript (A, B, C) within a row are significantly different (*p* < 0.05). 5′-CMP ^a^, 5-cytosine monophosphate; 5′-UMP, 5-uridine monophosphate; 5′-GMP, 5-guanosine monophosphate; 5′-AMP, 5-adenosine monophosphate. F ^b^, fresh shiitake mushroom; FD, freeze drying sample; HAD, hot-air drying sample; ND, natural drying sample. Flavor 5′-nucleotides ^c^: 5′-GMP + 5′-IMP + 5′-XMP, while only 5′-GMP was detected in this study. MSG-like 5′-nucleotide ^d^: 5′-AMP + 5′-GMP + 5′-IMP + 5′-XMP, while 5′-IMP and 5′-XMP was not detected in this study.

## Data Availability

Not applicable.
